# The pathogenesis of cardiac microlesion formation during severe bacteremic infection

**DOI:** 10.1371/journal.ppat.1009021

**Published:** 2020-11-19

**Authors:** Armand O. Brown, Danielle A. Garsin

**Affiliations:** Department of Microbiology and Molecular Genetics, The University of Texas Health Science Center at Houston, Houston, Texas, United States of America; Nanyang Technological University, SINGAPORE

## Cardiac microlesions are associated with a growing number of bacterial infections

Community acquired pneumonia (CAP) caused by the gram-positive bacterium *Streptococcus pneumoniae* has been shown to predispose older individuals (aged 65 years and older) to adverse long-term cardiac sequelae such as new or worsened heart failure and cardiac arrhythmia [1,2]. To better understand these clinical observations, investigators sought to determine the interactions of *S*. *pneumoniae* with the heart during invasive pneumococcal disease (IPD). As a result, *S*. *pneumoniae* was found to form bacteria-filled vacuoles (i.e., cardiac microlesions) within the ventricles of mice, nonhuman primates, and humans, leading to an immunoquiescent immune response, altered electrophysiology, cardiomyocyte cell death, and heart failure in a mouse model [2]. As such, the formation of microlesions, subsequent healing, and formation of myocardial fibrosis have been hypothesized to be a pathology that could partially explain the adverse cardiac events observed in patients hospitalized with CAP [2]. More recently, the human commensal gram-positive bacterium *Enterococcus faecalis* was also shown to promote cardiac microlesion formation in mice, while suppressing the host cardiomyocyte inflammatory response in vitro [3]. Similar observations of microlesion formation were made with the gram-negative bacterium *Franscisella tularensis* subspecies *novicida* [4] and gram-positive bacterium non-tuberculosis *Mycobacterium avium* [5]. Therefore, cardiac microlesion formation is becoming increasingly appreciated as a possible risk factor for adverse cardiac complications during severe bacterial infection [6]. This review seeks to shed light on these observations and the underlying mechanisms contributing to this newly described pathology.

## Cardiac microlesion may contribute to adverse cardiac events

Cardiac microlesions are characterized by the presence of bacteria-filled lesions largely devoid of immune cell infiltrate [2]. This is in contrast to myocardial abscesses, which are suppurative infections most commonly caused by *Staphylococcus aureus*, and that are characterized by robust immune cell infiltrate at sites of formation [2]. Pneumococcal cardiac microlesions are generally diffused across the ventricles and are associated with a suppressed immune response, altered electrophysiology, cardiomyocyte cell death, and cardiac failure in mice [2].

*E*. *faecalis* can cause a variety of severe infections, including bacteremia and infective endocarditis (IE) [7]. IE is an infection of the heart that involves the formation of vegetations on the surface of heart valves and inner chambers and has a 1-year mortality rate of approximately 29% [8]. To date, heart infections associated with *E*. *faecalis* have traditionally been characterized as occurring on the surfaces of the heart, rather than occurring within the myocardial tissue. Recently, *E*. *faecalis* was shown to cause cardiac microlesions during severe bacteremic infection [3]. Furthermore, *E*. *faecalis* triggered the cell death of cardiomyocytes following direct exposure in vitro and also induced apoptosis and necroptosis within microlesions in vivo [3].

In other studies, *Ft*.*n* was also shown to promote cardiac microlesion formation during severe infection of mice. *Ft*.*n* microlesions were associated with altered electrophysiology, cardiomyocyte cell death, increased production of inflammatory mediators in the blood, and myocardial inflammation [4]. Likewise, during severe infection of mice with non-tuberculosis *M*. *avium*, cardiac microlesions were observed to be associated with increased inflammatory mediators in the blood, altered electrophysiology, cardiac hypertrophy, premature atrial contraction, and cardiac dysrhythmia in old mice [5]. Although inflammatory mediators could be detected within the serum or hearts of mice infected with *Ft*.*n* and *M*. *avium*, the cardiac microlesion pathology observed is similar to those caused by *S*. *pneumoniae* and *E*. *faecalis*. These data suggest that the suppression of the immune response within the microenvironment of the myocardial tissue may be a common strategy used by bacterial pathogens that cause microlesions. Nonetheless, these observations highlight the need for further investigation in humans to determine the clinical significance.

## The virulence determinants affecting cardiac microlesion formation may differ between bacterial species

**Fig 1:**
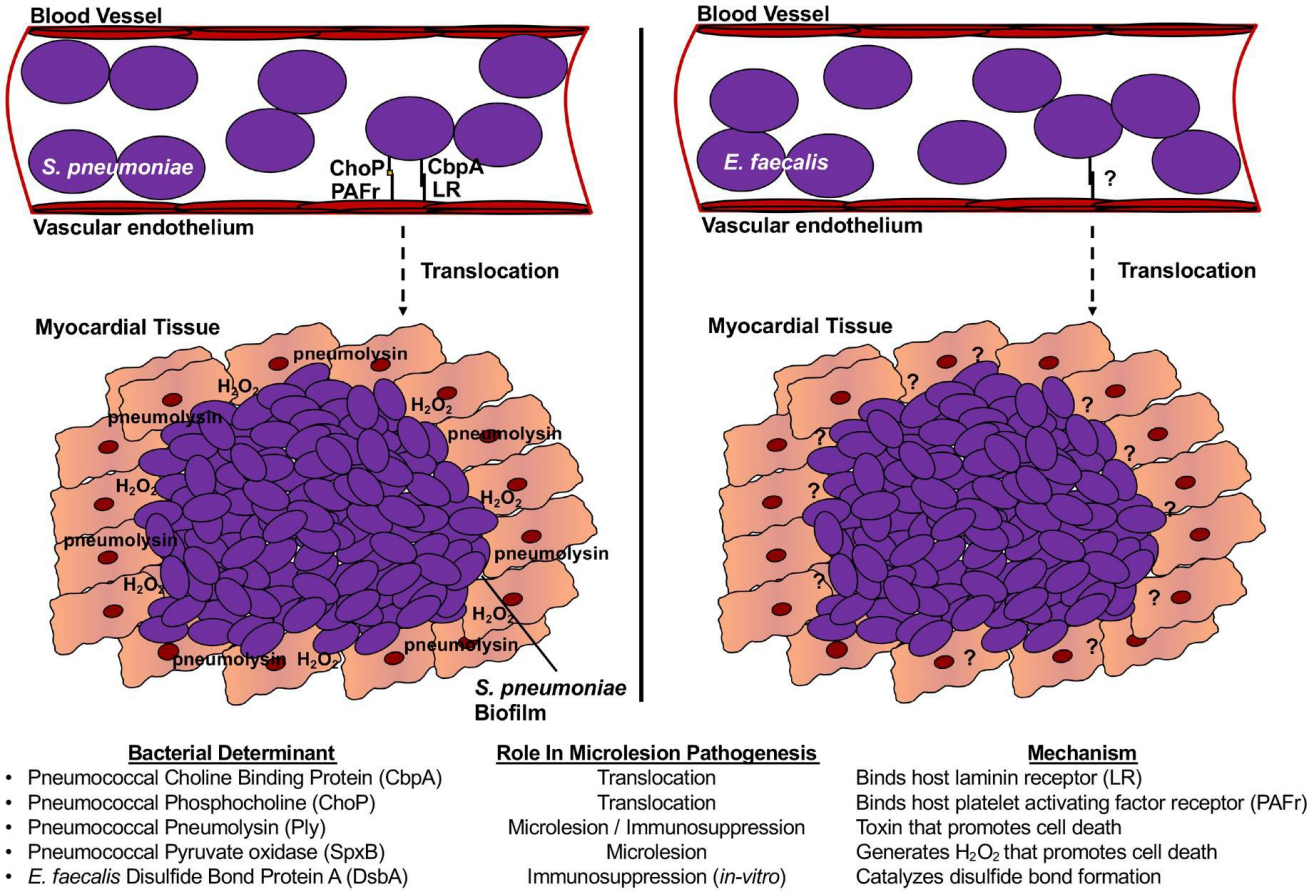
Virulence determinants known to contribute to microlesion formation. A model summarizing the known bacterial virulence determinants known to contribute to the formation of cardiac microlesion by facilitating vascular translocation, cell death, or immune suppression. CbpA, choline-binding protein A; ChoP, phosphocholine; DsbA, disulfide bond protein A; LR, laminin receptor; PAFr, platelet activating factor; Ply, pneumolysin; SpxB, pyruvate oxidase.

Bacterial factors that contribute to cardiac microlesion formation have been identified for *S*. *pneumoniae* and *E*. *faecalis*, but not yet for *M*. *avium or F*. *tularensis* (Fig 1). For instance, the pneumococcal virulence determinants choline-binding protein A (CbpA) and lipoteichoic acid–associated phosphocholine (ChoP) were shown to bind host laminin receptor (LR) and platelet activating factor (PAFr) on host endothelial cells (Fig 1), respectively. These critical interactions facilitate the translocation of the bacterium across the vascular endothelium and into the myocardium where the bacterium can replicate [2]. Subsequent studies focused on elucidating the mechanism of cardiomyocyte death determined that the pneumococcal cholesterol-dependent pore-forming toxin pneumolysin (Ply) and pyruvate oxidase (SpxB) generated hydrogen peroxide that induced the cell death of cardiomyocytes and infiltrating leukocytes [2,9,10].

Likewise, *E*. *faecalis* was also shown to invade the vascular endothelium in order to gain entry into the myocardial tissue where the bacterium could induce cell death [3]. Importantly, *E*. *faecalis* does not encode a homolog of the pneumococcal surface adhesin CbpA nor does it encode a homolog of pneumolysin (*ply*) suggesting that other factors may be involved. Moreover, *E*. *faecalis* does not encode a homolog of pneumococcal pyruvate oxidase (*spxB*). However, it can produce reactive oxygen species (ROS) [11]. As such, ROS secretion by *E*. *faecalis* might also contribute to cell death and microlesion formation. One protein identified as influencing *E*. *faecalis* cardiac microlesion formation is a disulfide bond-forming (Dsb) protein called DsbA. Thioredoxins such as DsbA have been implicated in various facets of bacterial fitness and pathogenicity such as biofilm formation, cell division, virulence, cell motility, cell wall synthesis, and growth. These proteins, which are characterized by their conserved CXXC active-site motif, function by interacting with the free thiol groups of substrate cysteines, leading to the catalysis of a disulfide bond. While much of our understanding comes from the study of oxidative protein folding in gram-negatives, less is understood with regard to gram-positive bacteria [12].

In recent work investigating the enzymes that contribute to the posttranslational processing of the *E*. *faecalis* bacteriocin EntV, researchers identified the previously uncharacterized thioredoxin DsbA as a requirement for the activity of EntV [13]. Further characterization showed that DsbA also contributes to *E*. *faecalis* virulence and cardiac microlesion formation, suggesting a deeper role for this thioredoxin in *E*. *faecalis* pathogenesis [3]. Considering that DsbA proteins generally facilitate extracytoplasmic protein disulfide bond formation, it is hypothesized that DsbA substrates that contribute to *E*. *faecalis* pathogenesis are either secreted or cell surface factors. In contrast to *E*. *faecalis*, *S*. *pneumoniae*, *Ft*.*n*, and *M*. *avium* do not encode a *dsbA* homolog, thus DsbA’s influence on microlesion formation may be through a mechanism unique to *E*. *faecalis*. Further investigation and identification of *E*. *faecalis* DsbA substrates will be critical to our understanding of DsbA’s role in modulating pathogenesis.

## Bacteria that form cardiac microlesions can subvert the immune response

Cardiac microlesions are characterized by bacteria-filled vacuoles, cell death, and a dampened immune response [2]. Studies aimed at deciphering these observations determined that *S*. *pneumoniae* forms biofilms within cardiac microlesions resulting in the enhanced secretion of pneumolysin relative to planktonic-grown pneumococci [9]. As a result, pneumolysin kills resident cardiac macrophages, leading to subversion of the cytokine/chemokine response and immune cell infiltration [9]. While *E*. *faecalis* has been observed to form biofilms on the inner surfaces of heart such as heart valves during IE [8], it is unclear if the bacteria aggregated within microlesions are in the form of biofilms. Likewise, while *M*. *avium* and *Ft*.*n* have also been observed to form biofilms on biotic and abiotic surfaces [14,15], it is unclear if they are forming biofilms within microlesions. In addition to biofilms, further investigations have revealed that infiltrated macrophages die as a result of necroptosis mediated by pneumolysin [16]. *E*. *faecalis* has long been known to subvert the host immune response as well. In a recent study, phagocytosed *E*. *faecalis* was shown to survive within mouse peritoneal macrophages through the inhibition of apoptosis [17] and through resistance of phagosomal acidification and autophagy [18]. *Ft*.*n* and *M*. *avium* are intracellular pathogens that can readily evade macrophage killing through a number of mechanisms involving inhibition of phagocytosis, inhibition of phagosomal acidification, suppression of macrophage ROS, avoidance of inflammatory signaling, and degradation, among others [19,20]. Interestingly, *E*. *faecalis* infection of cardiomyocytes in vitro promoted an immunoquiescent immune response that appears to be dependent on the presence of *dsbA*, suggesting an immunomodulatory role for DsbA [3]. Taken together, these data highlight the importance of bacterial immune evasion during the formation of cardiac microlesions.

## Cardiac microlesion formation requires further study

The emerging field of cardiac microlesion formation has not only yielded interesting insights into the impact of their formation on the host but also on the mechanisms used by the bacteria to promote their formation. In the few years since the original observation of cardiac microlesion formation during IPD, other investigators have shown that additional bacterial pathogens are capable of causing cardiac microlesions. These important observations suggest that cardiac microlesion formation may be more widespread than was previously appreciated and may occur through diverse mechanisms. Future studies aimed at investigating whether these other agents also cause human heart infections and identifying the bacterial and host factors involved will be critical toward understanding the impact of severe bacteremic infections on the heart and the long-term consequences.

## References

[1] Corrales-Medina VF, Suh KN, Rose G, Chirinos JA, Doucette S, Cameron DW, et al Cardiac complications in patients with community-acquired pneumonia: a systematic review and meta-analysis of observational studies. PLoS Med. 2011;8(6):e1001048 Epub 2011 Jul 9. 10.1371/journal.pmed.1001048 21738449 PMC3125176

[2] Brown AO, Mann B, Gao G, Hankins JS, Humann J, Giardina J, et al Streptococcus pneumoniae translocates into the myocardium and forms unique microlesions that disrupt cardiac function. PLoS Pathog. 2014;10(9):e1004383 Epub 2014 Sep 19. 10.1371/journal.ppat.1004383 25232870 PMC4169480

[3] Brown AO, Singh KV, Cruz MR, Kaval KG, Francisco LE, Murray BE, et al Cardiac Microlesions Form During Severe Bacteremic Enterococcus faecalis Infection. J Infect Dis. 2020 Epub 2020 Jul 1. 10.1093/infdis/jiaa371 . 32597945 PMC7881331

[4] Makara MA, Hoang KV, Ganesan LP, Crouser ED, Gunn JS, Turner J, et al Cardiac Electrical and Structural Changes During Bacterial Infection: An Instructive Model to Study Cardiac Dysfunction in Sepsis. J Am Heart Assoc. 2016;5(9). Epub 2016 Sep 14. 10.1161/JAHA.116.003820 27620887 PMC5079037

[5] Headley CA, Gerberick A, Mehta S, Wu Q, Yu L, Fadda P, et al Nontuberculous mycobacterium M. avium infection predisposes aged mice to cardiac abnormalities and inflammation. Aging Cell. 2019;18(3):e12926 Epub 2019 Mar 6. 10.1111/acel.12926 30834643 PMC6516181

[6] Musher DM, Abers MS, Corrales-Medina VF. Acute Infection and Myocardial Infarction. N Engl J Med. 2019;380(2):171–6. Epub 2019 Jan 10. 10.1056/NEJMra1808137 . 30625066

[7] Fiore E, Van Tyne D, Gilmore MS. Pathogenicity of Enterococci. Microbiol Spectr. 2019;7(4). Epub 2019 Jul 13. 10.1128/microbiolspec.GPP3-0053-2018 31298205 PMC6629438

[8] Chirouze C, Athan E, Alla F, Chu VH, Ralph Corey G, Selton-Suty C, et al Enterococcal endocarditis in the beginning of the 21st century: analysis from the International Collaboration on Endocarditis-Prospective Cohort Study. Clin Microbiol Infect. 2013;19(12):1140–7. Epub 2013 Mar 23. 10.1111/1469-0691.12166 . 23517406

[9] Shenoy AT, Brissac T, Gilley RP, Kumar N, Wang Y, Gonzalez-Juarbe N, et al Streptococcus pneumoniae in the heart subvert the host response through biofilm-mediated resident macrophage killing. PLoS Pathog. 2017;13(8):e1006582 Epub 2017 Aug 26. 10.1371/journal.ppat.1006582 28841717 PMC5589263

[10] Brissac T, Shenoy AT, Patterson LA, Orihuela CJ. Cell Invasion and Pyruvate Oxidase-Derived H2O2 Are Critical for Streptococcus pneumoniae-Mediated Cardiomyocyte Killing. Infect Immun. 2018;86(1). Epub 2017 Oct 25. 10.1128/IAI.00569-17 29061707 PMC5736805

[11] Wang X, Huycke MM. Extracellular superoxide production by Enterococcus faecalis promotes chromosomal instability in mammalian cells. Gastroenterology. 2007;132(2):551–61. Epub 2007 Jan 30. 10.1053/j.gastro.2006.11.040 . 17258726

[12] Reardon-Robinson ME, Ton-That H. Disulfide-Bond-Forming Pathways in Gram-Positive Bacteria. J Bacteriol. 2015;198(5):746–54. 10.1128/JB.00769-15 26644434 PMC4810614

[13] Brown AO, Graham CE, Cruz MR, Singh KV, Murray BE, Lorenz MC, et al Antifungal activity of the Enterococcus faecalis peptide EntV requires protease cleavage and disulfide bond formation. MBio. 2019;10(4):1–13. Epub 2019 Jul/Aug. e01334-19. 10.1128/mBio.01334-19 31266876 PMC6606811

[14] Rose SJ, Bermudez LE. Mycobacterium avium biofilm attenuates mononuclear phagocyte function by triggering hyperstimulation and apoptosis during early infection. Infect Immun. 2014;82(1):405–12. Epub 2013 Nov 6. 10.1128/IAI.00820-13 24191301 PMC3911830

[15] Margolis JJ, El-Etr S, Joubert LM, Moore E, Robison R, Rasley A, et al Contributions of Francisella tularensis subsp. novicida chitinases and Sec secretion system to biofilm formation on chitin. Appl Environ Microbiol. 2010;76(2):596–608. Epub 2009 Dec 2. 10.1128/AEM.02037-09 19948864 PMC2805214

[16] Gilley RP, Gonzalez-Juarbe N, Shenoy AT, Reyes LF, Dube PH, Restrepo MI, et al Infiltrated Macrophages Die of Pneumolysin-Mediated Necroptosis following Pneumococcal Myocardial Invasion. Infect Immun. 2016;84(5):1457–69. Epub 2016 Mar 2. 10.1128/IAI.00007-16 26930705 PMC4862731

[17] Zou J, Shankar N. Enterococcus faecalis infection activates phosphatidylinositol 3-kinase signaling to block apoptotic cell death in macrophages. Infect Immun. 2014;82(12):5132–42. Epub 2014 Oct 1. 10.1128/IAI.02426-14 25267834 PMC4249289

[18] Zou J, Shankar N. The opportunistic pathogen Enterococcus faecalis resists phagosome acidification and autophagy to promote intracellular survival in macrophages. Cell Microbiol. 2016;18(6):831–43. Epub 2015 Dec 15. 10.1111/cmi.12556 . 26663775

[19] Steiner DJ, Furuya Y, Metzger DW. Host-pathogen interactions and immune evasion strategies in Francisella tularensis pathogenicity. Infect Drug Resist. 2014;7:239–51. Epub 2014 Sep 27. 10.2147/IDR.S53700 25258544 PMC4173753

[20] Early J, Fischer K, Bermudez LE. Mycobacterium avium uses apoptotic macrophages as tools for spreading. Microb Pathog. 2011;50(2):132–9. Epub 2010 Dec 21. 10.1016/j.micpath.2010.12.004 21167273 PMC3030681

